# Physical Exercise and Nutritional Approaches on Gastrointestinal Pathophysiology

**DOI:** 10.3390/nu18182998

**Published:** 2026-09-14

**Authors:** Francisco Leonardo Torres-Leal, José Luiz de Brito Alves, Moisés Tolentino Bento da Silva

**Affiliations:** 1 Metabolic Diseases, Exercise and Nutrition Research Group (DOMEN), Laboratory of Metabolic Diseases Glauto Tuquarre, Department of Biophysics and Physiology, Center for Health Sciences, Federal University of Piauí, Teresina 64049-550, PI, Brazil; torresleal@ufpi.edu.br; 2 Department of Nutrition, Health Sciences Center, Federal University of Paraiba, João Pessoa 58051-900, PB, Brazil; jose.luiz@academico.ufpb.br; 3 Laboratory of Physiology, RISE-Health: Health Research Network, Department of Immuno-Physiology and Pharmacology, School of Medicine and Biomedical Science, University of Porto (ICBAS-UP), 4050-313 Porto, Portugal

## Introduction

The practice of different types of sports involves a workload that is prescribed based on volume and intensity. These can include longer-duration activities, such as marathons and cycling competitions, that affect gastrointestinal tract (GIT) function and can lead to gastrointestinal disorders. However, studies focus only on describing the effect, leaving the mechanisms still unknown. The redistribution of blood flow is one hypothesis because it induces gastrointestinal ischemia, alters mechanical forces, changes the microbiota, and results in injury to gastrointestinal mucosal activity, neuroendocrine modification, and stress [1]. It is estimated that 30–90% of distance runners experience gastrointestinal symptoms, such as vomiting, nausea, heartburn, gastroesophageal reflux, diarrhea, bleeding from the stools, and changes in bowel movements [2]. High-intensity exercise decreases gastric emptying, a phenomenon associated with lactate-induced acidemia [3]. On the other hand, low-intensity exercise, such as walking (at 28, 41, and 56% of VO_2_ max) and running (at 57 and 65% VO_2_ max), accelerates it [4].

Regarding sports nutrition, various factors can influence the prevalence and incidence of GI symptoms during exercise. These factors include the intake of different carbohydrate (CHO) concentrations and food types pre- and post-exercise; osmolality and acidity; dietary intake of fiber, protein, and fat; and changes in the intestinal environment [5]. Understanding the role of the microbiota is essential, and modulating it involves changes to metabolic pathways, including nutrient digestion, metabolic processes, immune system regulation, and preservation of intestinal epithelial tissue development. In this Special Issue of *Nutrients*, entitled “Physical Exercise and Nutritional Approaches on Gastrointestinal Pathophysiology,” the authors presented a collection of papers evaluating the effects of physical exercise and nutrition in health and illness.

In their study, Lopes et al. investigate the effects of acute administration of olive, linseed, soybean, and palm oils on gastrointestinal motility, satiety, and appetite, as well as on their modulation via the vagus nerve. These authors found that different oils have distinct effects on parameters related to food intake. In this sense, olive oil reduces food intake, thereby affecting the gastrointestinal tract and making it a recommended adjunct for weight loss. On the other hand, palm oil intake inhibits food intake in the absence of an autonomic response. Still, it is not advised, due to its contribution to the development of cardiometabolic disorders. (Contribution 1).

The pediatric population presents with a range of gastrointestinal problems. Small intestinal bacterial overgrowth (SIBO) in children was studied by Costa et al., who assessed anthropometric, biochemical, and inflammatory parameters, as well as dietary intake, in Brazilian children diagnosed with SIBO, and compared these measures with those of their counterparts without SIBO. The authors showed a 13% prevalence of SIBO among children in northeastern Brazil. They showed that consumption of ultra-processed foods and serum IL-6 levels may influence its occurrence in the pediatric population. (Contribution 2).

In (Contribution 3), Lietzén et al. investigate the effects of obesity on insulin-stimulated intestinal function from blood (small intestine and colon), microbiota composition in monozygotic twins with different body mass index (BMI), and how these alterations can be modified with chronic regular physical exercise. In this sense, the authors observed that it impairs insulin-stimulated intestinal uptake of circulating glucose from blood, independent of genetic factors. On the other hand, regular physical exercise improved aerobic fitness and whole-body insulin sensitivity in both the leaner and the heavier co-twins. Although both twin groups exhibited some genus-level microbiota changes, most changes in insulin-stimulated colon and microbiota composition were significant in the leaner twins. In another study, Bianco et al. investigated the impact of a 12-week exercise program (Fitwalking) on the health of intestinal barrier integrity in patients with intestinal bowel syndrome. The authors showed that regular physical exercise improves intestinal barrier integrity, which is more impaired in patients with obesity who have intestinal bowel syndrome. Moreover, the authors observed changes in gut barrier integrity and in the severity of gastrointestinal injury by monitoring biomarkers and proteins, such as zonulin. This human protein regulates intestinal barrier permeability by controlling tight junctions between intestinal cells and by regulating intestinal fatty acid-binding protein (I-FABP) levels. However, the authors suggested that future studies should explore longer intervention durations and larger populations, and investigate the mechanisms underlying exercise-induced changes in intestinal permeability and gut health markers. (Contribution 4).

The Mediterranean diet is widely recognized for its health benefits, as it modulates the composition of the gut microbiota and reduces the risk of metabolic, cardiovascular, and neurodegenerative disorders. A high intake of plant-based foods, monounsaturated fats, polyphenols and extra-virgin olive oil characterizes it. In the review by Perrone et al. (Contribution 5), after examining various studies, the authors suggest that the Mediterranean diet is a powerful tool for modulating the gut microbiota, has positive effects on metabolic and cardiovascular parameters, and can improve cognitive health. Moreover, the combination of fibers, healthy fats, polyphenols, and plant-based oils plays a crucial role in promoting the growth of beneficial gut microbiota.

In their study, Álvarez-Herms et al. (Contribution 6) investigate how regulating sensations of fatigue and discomfort during maximal endurance exercise is crucial for making informed decisions about persistence or failure. In this sense, the focus is on the possible relationship between changes in the balance of the gut microbiota and its association with the nervous system, in terms of the adaptive response in chronic fatigue. In this sense, substantial evidence suggests that the gut microbiota can rapidly adapt to afferences from the brain axis, with a possible role in modulating the sensations of fatigue, pain, and discomfort. Therefore, the host–microbiota relationship could determine predisposition to better or worse performance during endurance exercise by increasing the perceived and tolerated thresholds. A richer and more diverse gut microbiota in athletes, compared with that of sedentary subjects, can improve the levels of bacteria-derived metabolites associated with fatigue-related brain activity.

In the study on digestive vulnerability and exercise exposure as correlates of gastrointestinal symptoms, Mauvieux et al. investigate whether digestive vulnerability expressed during training and cumulative exercise exposure are associated with gastrointestinal symptoms during competition and race withdrawal in endurance and ultra-endurance athletes. The authors’ results suggest that gastrointestinal symptoms and race withdrawal in endurance athletes are more associated with digestive vulnerability observed during training and cumulative exercise exposure than with nutritional parameters. These findings support a vulnerability–exposure framework in which individual digestive susceptibility interacts with prolonged physiological stress during endurance exercise (Contribution 7).

Alsinani et al. conducted a review of exercise and the gut microbiome, including potential mechanisms and clinical applications. The gut microbiome is an essential regulator of host metabolism, immunity function, and the gut–brain axis. Physical exercise is a non-pharmacological treatment that can modulate microbiota behavior. However, human evidence on the possible mechanisms remains heterogeneous, and the translational gap persists. In this review, the authors indicate that physical exercise modulates the gut microbiome and improves metabolic outcomes. However, evidence linking exercise-induced microbial shifts to enhanced athletic performance in humans remains lacking. Thus, the authors indicate possible limitations and suggest future research involving diet-controlled randomized controlled trials with longer intervention durations, shotgun omics-based assessments, and mechanistic validation of the gut–muscle axis in humans. (Contribution 8).

In conclusion, this Special Issue presents a range of high-quality studies that advance knowledge in the field of exercise and nutrition as they relate to gastrointestinal pathophysiology.
